# Supplementary material for: Oligomeric HIV-1 Integrase Structures Reveal Functional Plasticity for Intasome Assembly and RNA Binding
Source: bioRxiv. 2024 Jan 27:2024.01.26.577436. Preprint. [Version 1] doi: 10.1101/2024.01.26.577436 (PMC10849644; doi:10.1101/2024.01.26.577436)

bioRxiv preprint doi: https://doi.org/10.1101/2024.01.26.577436; this version posted January 27, 2024. The copyright holder for this preprint (which was not certified by peer review) is the author/funder, who has granted bioRxiv a license to display the preprint in perpetuity. It is made available under aCC-BY 4.0 International license.

Cryo-EM data collection, refinement and validation statistics

	IN-IBD complex (EMDB-XXXX) (PDB-XXXX)	Crosslinked WT Intasome (EMDB-XXXX) (PDB-XXXX)
**Data collection and processing**		
Magnification	105,000x	29,000
Voltage (kV)	300	300
Electron exposure (e–/Å2)	49.5	60.0
Defocus range (μm)	-0.8 to -2.2	-1.0 to -3.0
Pixel size (Å)	0.83	1.015
Symmetry imposed	C2	C2 → C1
Number of particles (in final map).	340,383	11,658
Map resolution (Å)	3.0	5.9
FSC threshold	0.143	0.143
Map resolution range (Å)	3.0 – 4.0	3.0 – 12.0
**Refinement**		
Initial model used (PDB codes)	1ITG, 1IHV, 1WJA	
Model resolution (Å)	4.0	
FSC threshold	0.5	
Model resolution range (Å)	3.5 – 4.5	
Map sharpening *B* factor ($Å^2$)	-140	
Model composition		
Non-hydrogen atoms	11,176	
Protein residues	1,392	
B factors ($Å^2$)		
Protein	92	
R.m.s. deviations		
Bond lengths (Å)	0.007	
Bond angles (°)	1.007	
Validation		
MolProbity score	2.4	
Clashscore	14.9	
Poor rotamers (%)	2.3	
Ramachandran plot		
Favored (%)	93.1	
Allowed (%)	6.8	
Disallowed (%)	0.1	

Supplementary Table 1. Cryo-EM data collection, refinement, and validation statistics.

bioRxiv preprint doi: https://doi.org/10.1101/2024.01.26.577436; this version posted January 27, 2024. The copyright holder for this preprint (which was not certified by peer review) is the author/funder, who has granted bioRxiv a license to display the preprint in perpetuity. It is made available under aCC-BY 4.0 International license.

Supplementary Figure 1

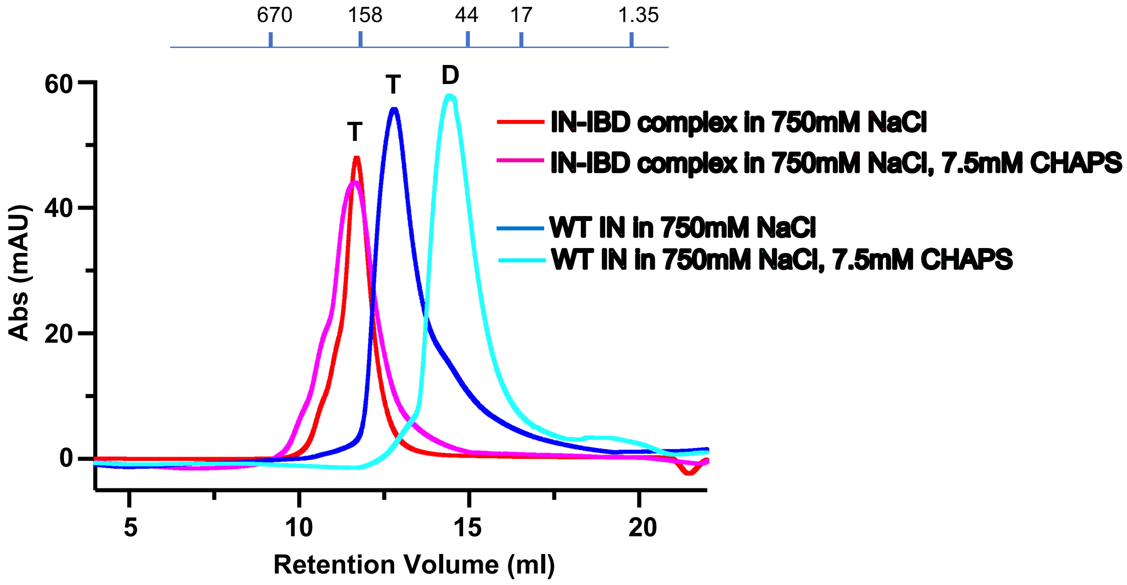

bioRxiv preprint doi: https://doi.org/10.1101/2024.01.26.577436; this version posted January 27, 2024. The copyright holder for this preprint (which was not certified by peer review) is the author/funder, who has granted bioRxiv a license to display the preprint in perpetuity. It is made available under aCC-BY 4.0 International license.

Supplementary Figure 2

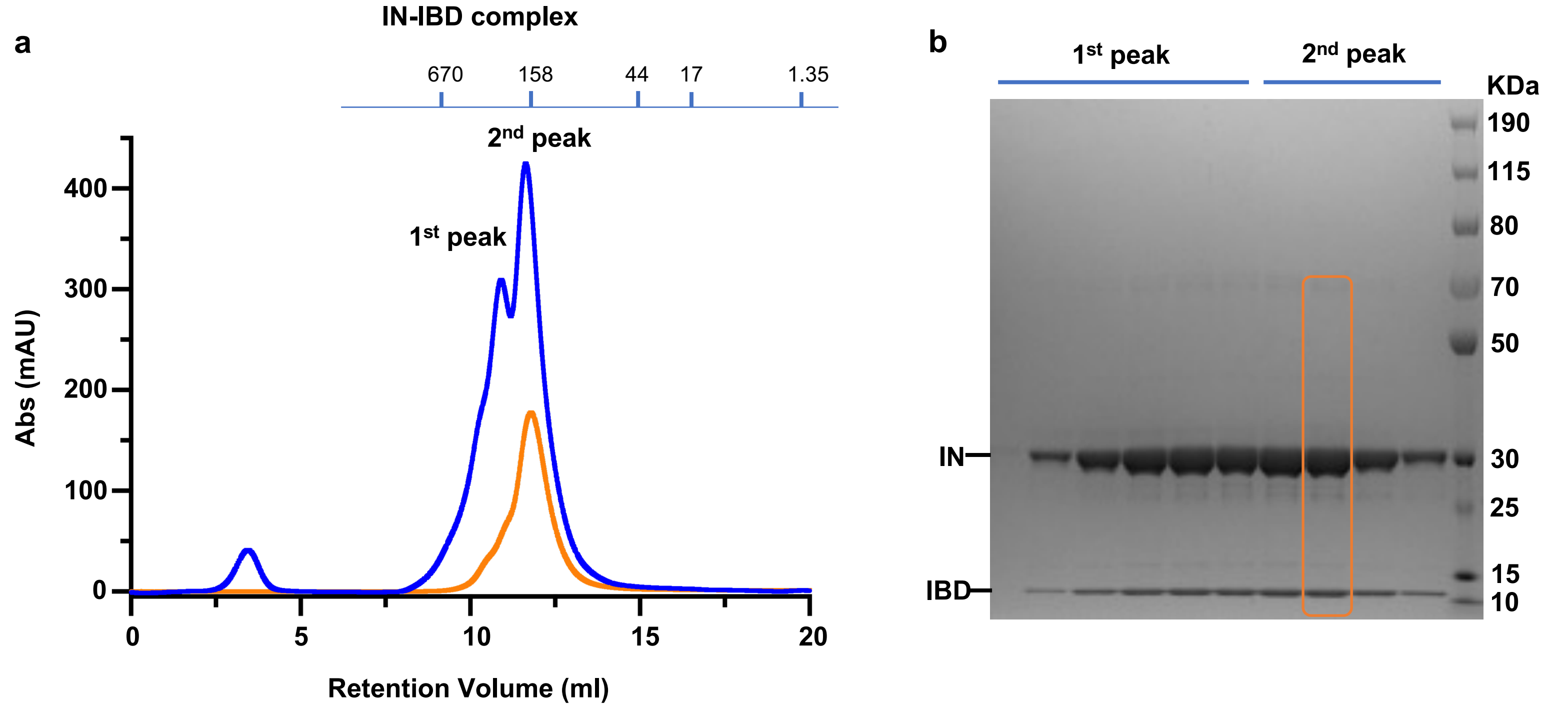

bioRxiv preprint doi: https://doi.org/10.1101/2024.01.26.577436; this version posted January 27, 2024. The copyright holder for this preprint (which was not certified by peer review) is the author/funder, who has granted bioRxiv a license to display the preprint in perpetuity. It is made available under aCC-BY 4.0 International license.

Supplementary Figure 3

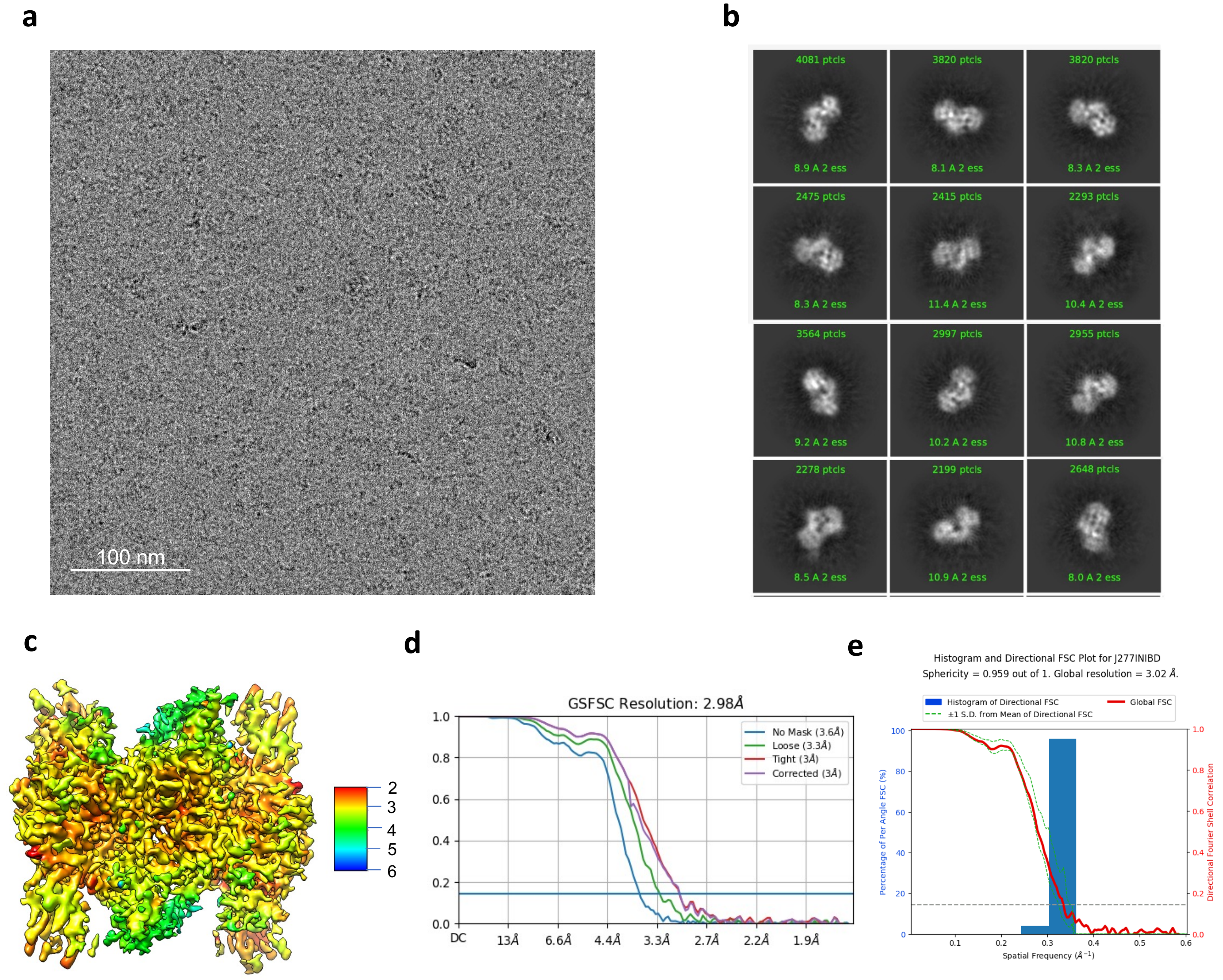

bioRxiv preprint doi: https://doi.org/10.1101/2024.01.26.577436; this version posted January 27, 2024. The copyright holder for this preprint (which was not certified by peer review) is the author/funder, who has granted bioRxiv a license to display the preprint in perpetuity. It is made available under aCC-BY 4.0 International license.

Supplementary Figure 4

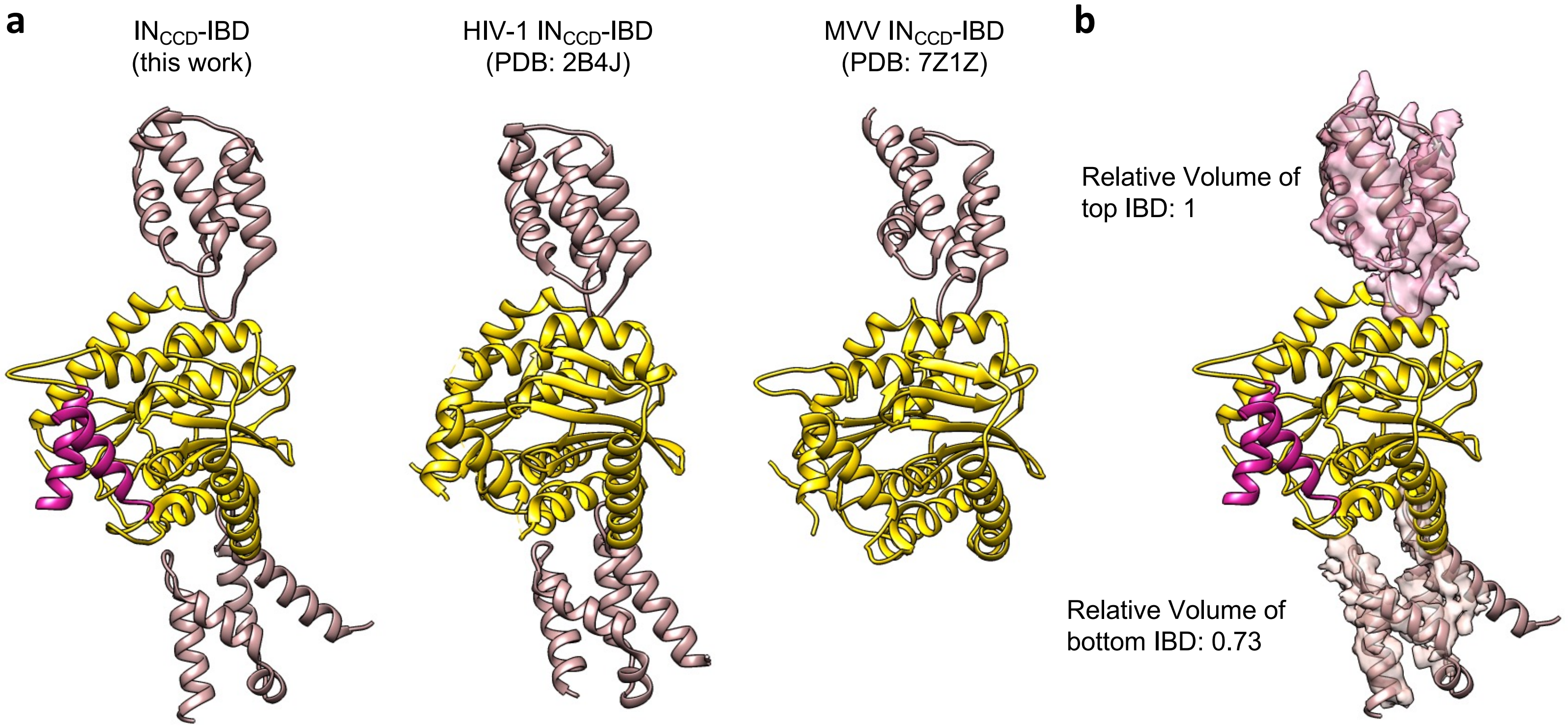

bioRxiv preprint doi: https://doi.org/10.1101/2024.01.26.577436; this version posted January 27, 2024. The copyright holder for this preprint (which was not certified by peer review) is the author/funder, who has granted bioRxiv a license to display the preprint in perpetuity. It is made available under aCC-BY 4.0 International license.

Supplementary Figure 5

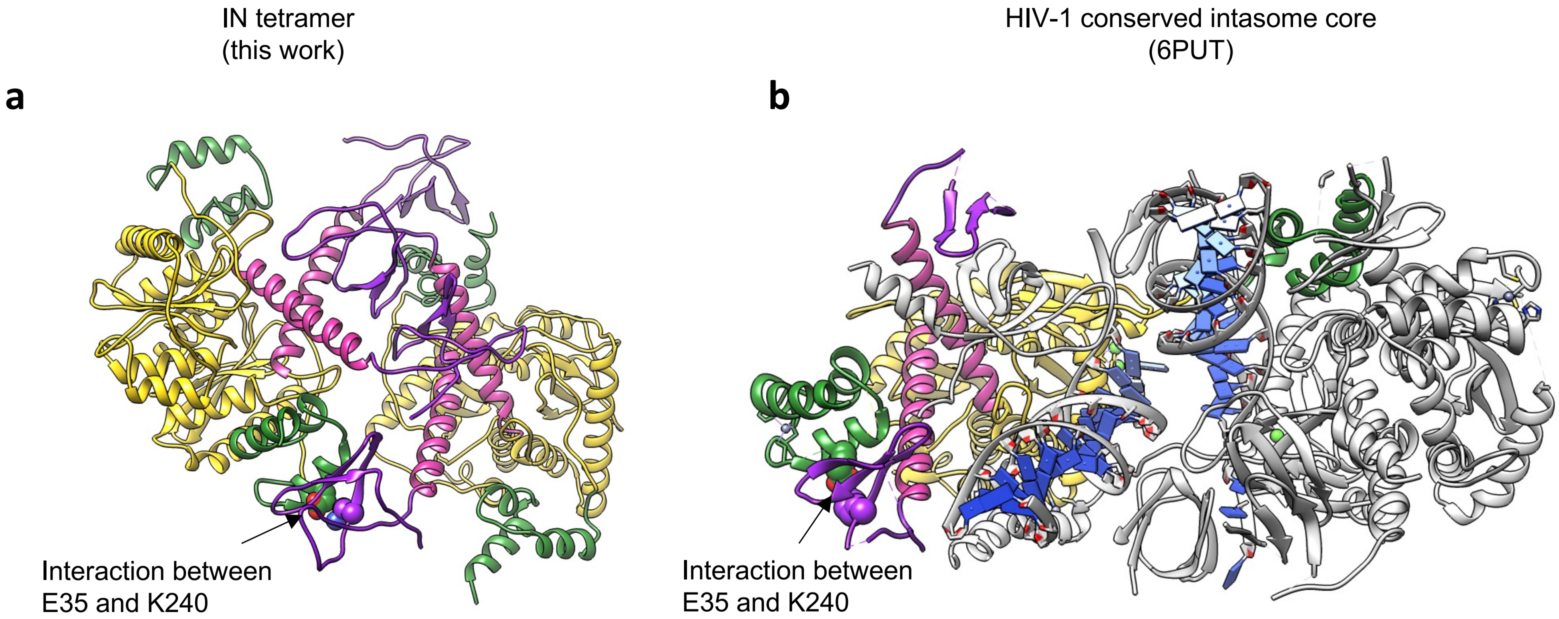

bioRxiv preprint doi: https://doi.org/10.1101/2024.01.26.577436; this version posted January 27, 2024. The copyright holder for this preprint (which was not certified by peer review) is the author/funder, who has granted bioRxiv a license to display the preprint in perpetuity. It is made available under aCC-BY 4.0 International license.

Supplementary Figure 6

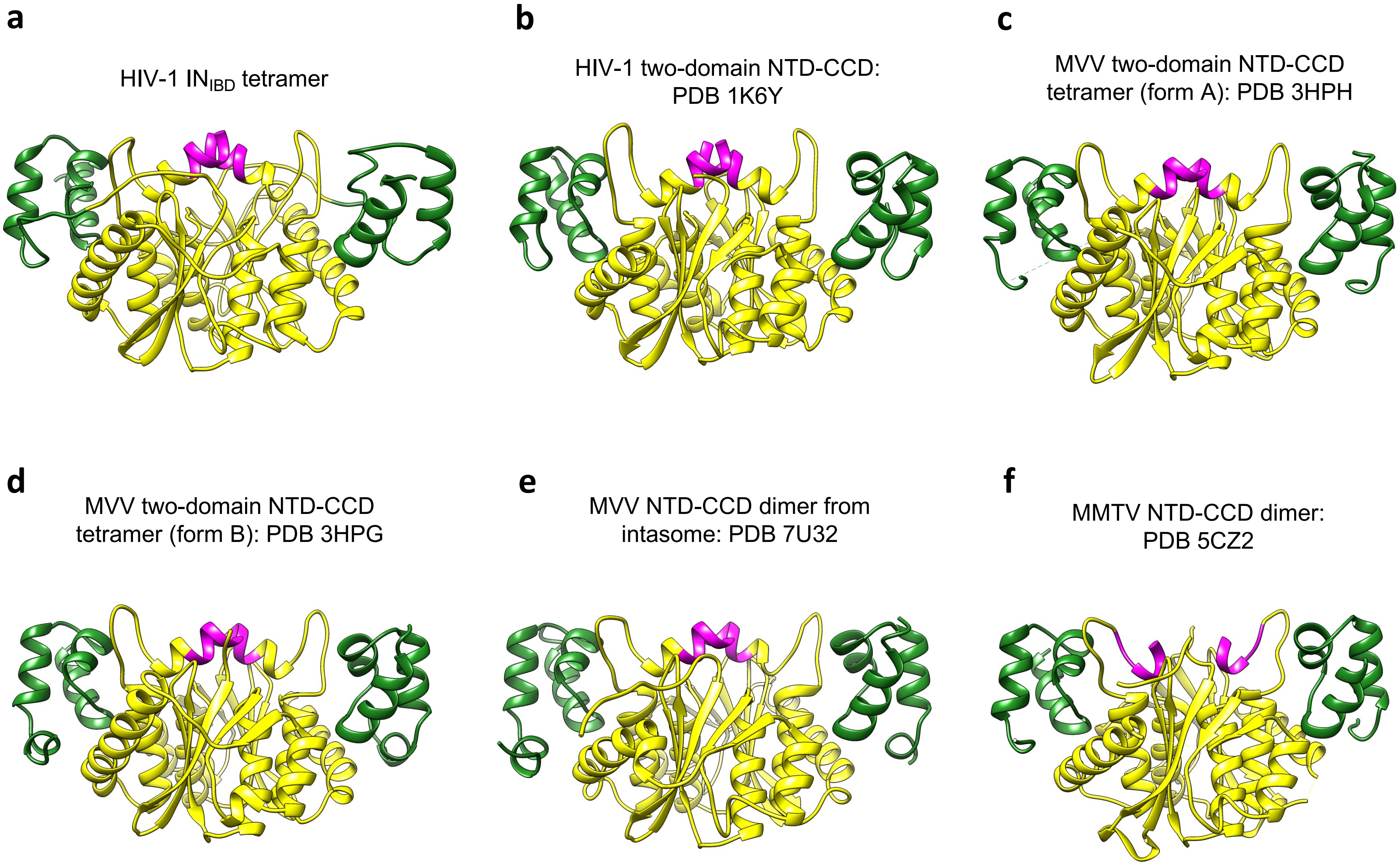

bioRxiv preprint doi: https://doi.org/10.1101/2024.01.26.577436; this version posted January 27, 2024. The copyright holder for this preprint (which was not certified by peer review) is the author/funder, who has granted bioRxiv a license to display the preprint in perpetuity. It is made available under aCC-BY 4.0 International license.

Supplementary Figure 7

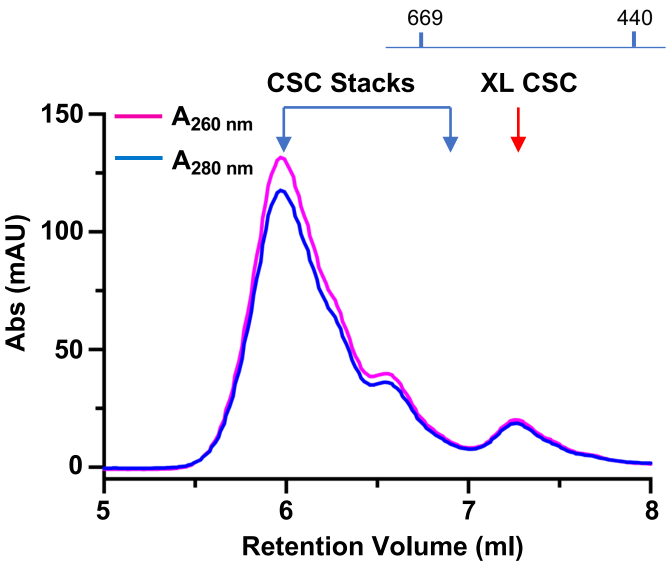

bioRxiv preprint doi: https://doi.org/10.1101/2024.01.26.577436; this version posted January 27, 2024. The copyright holder for this preprint (which was not certified by peer review) is the author/funder, who has granted bioRxiv a license to display the preprint in perpetuity. It is made available under aCC-BY 4.0 International license.

Supplementary Figure 8

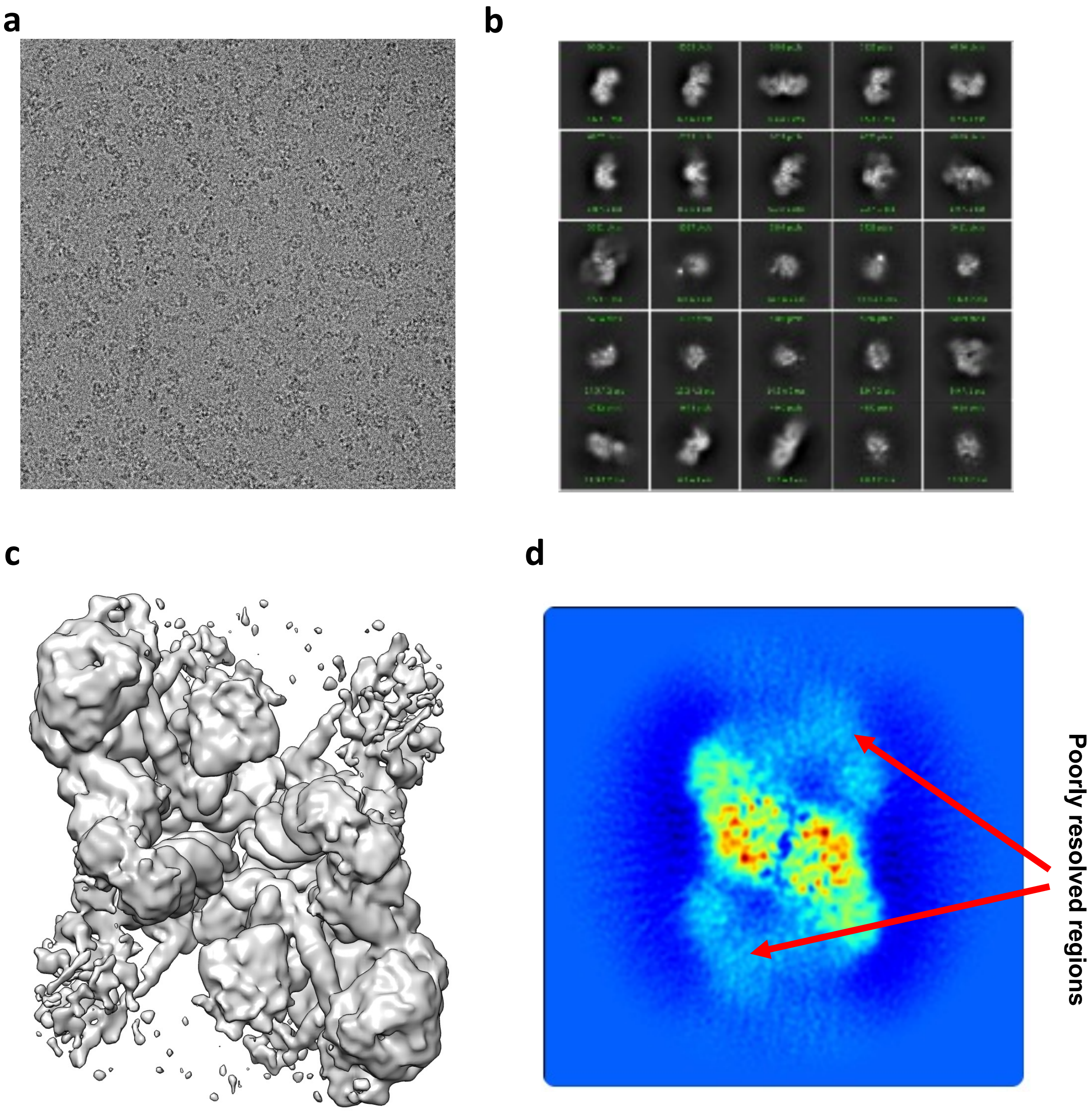

bioRxiv preprint doi: https://doi.org/10.1101/2024.01.26.577436; this version posted January 27, 2024. The copyright holder for this preprint (which was not certified by peer review) is the author/funder, who has granted bioRxiv a license to display the preprint in perpetuity. It is made available under aCC-BY 4.0 International license.

Supplementary Figure 9

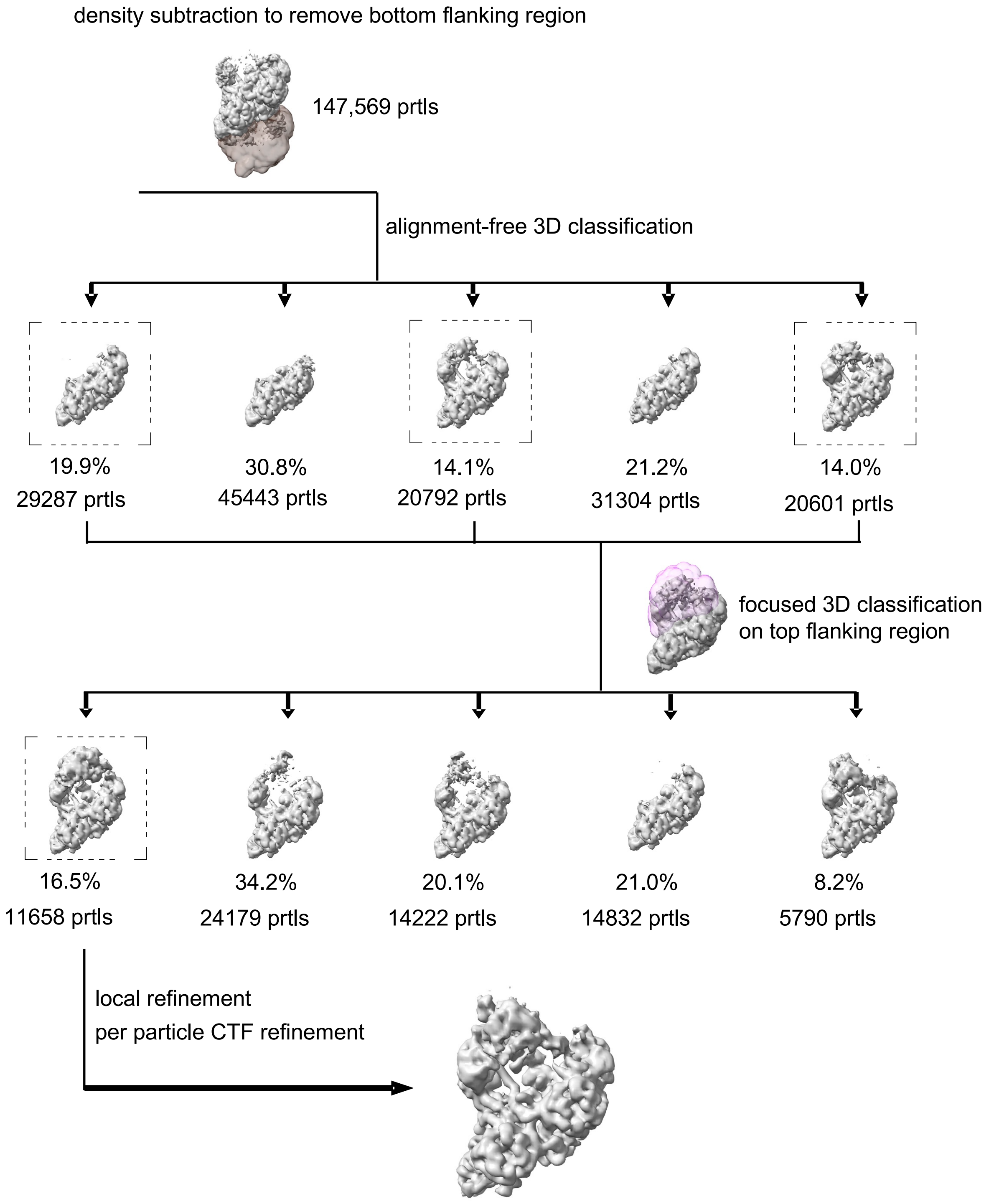

Supplementary Figure 10

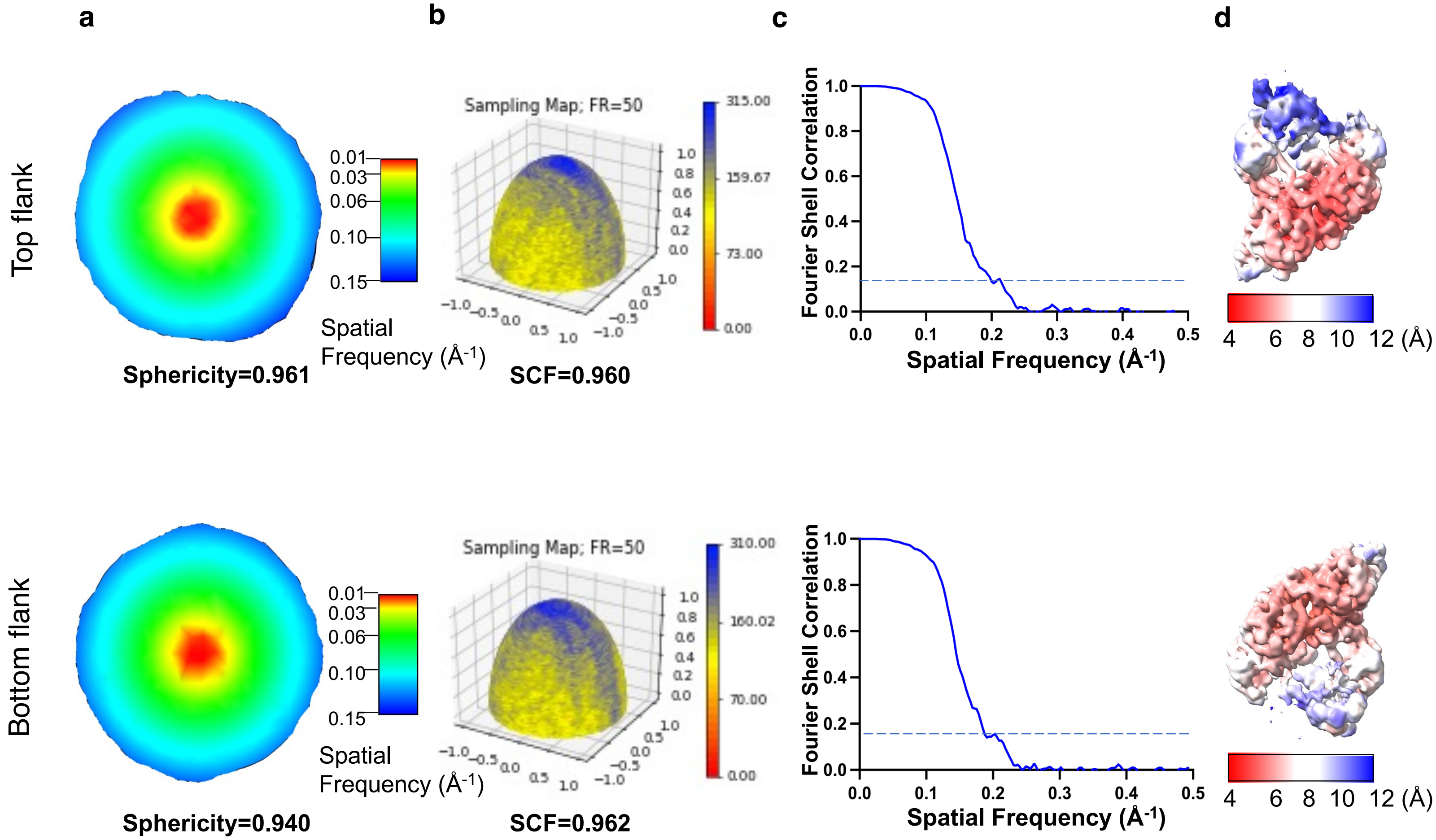

Supplementary Figure 11

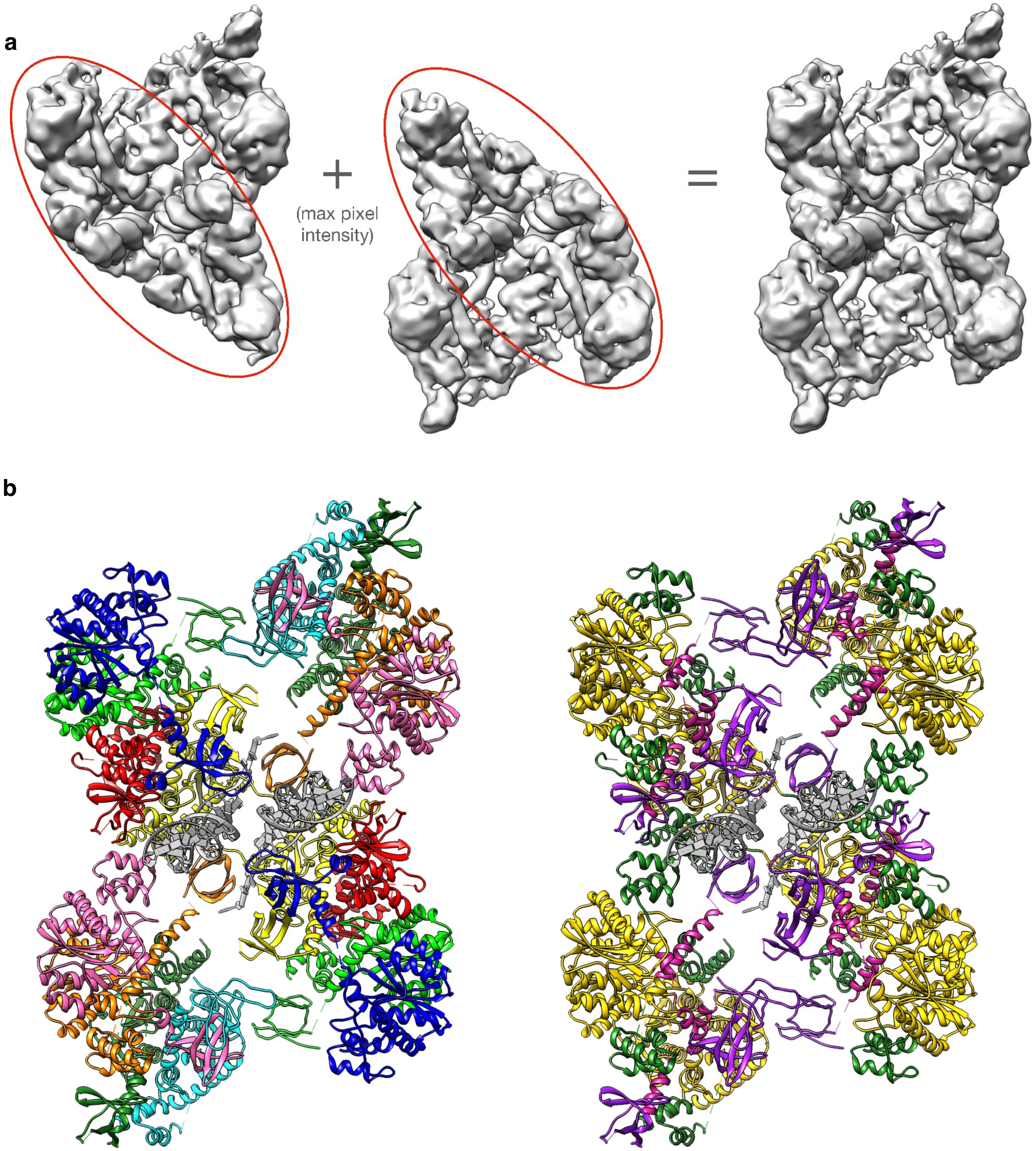

Supplementary Figure 12

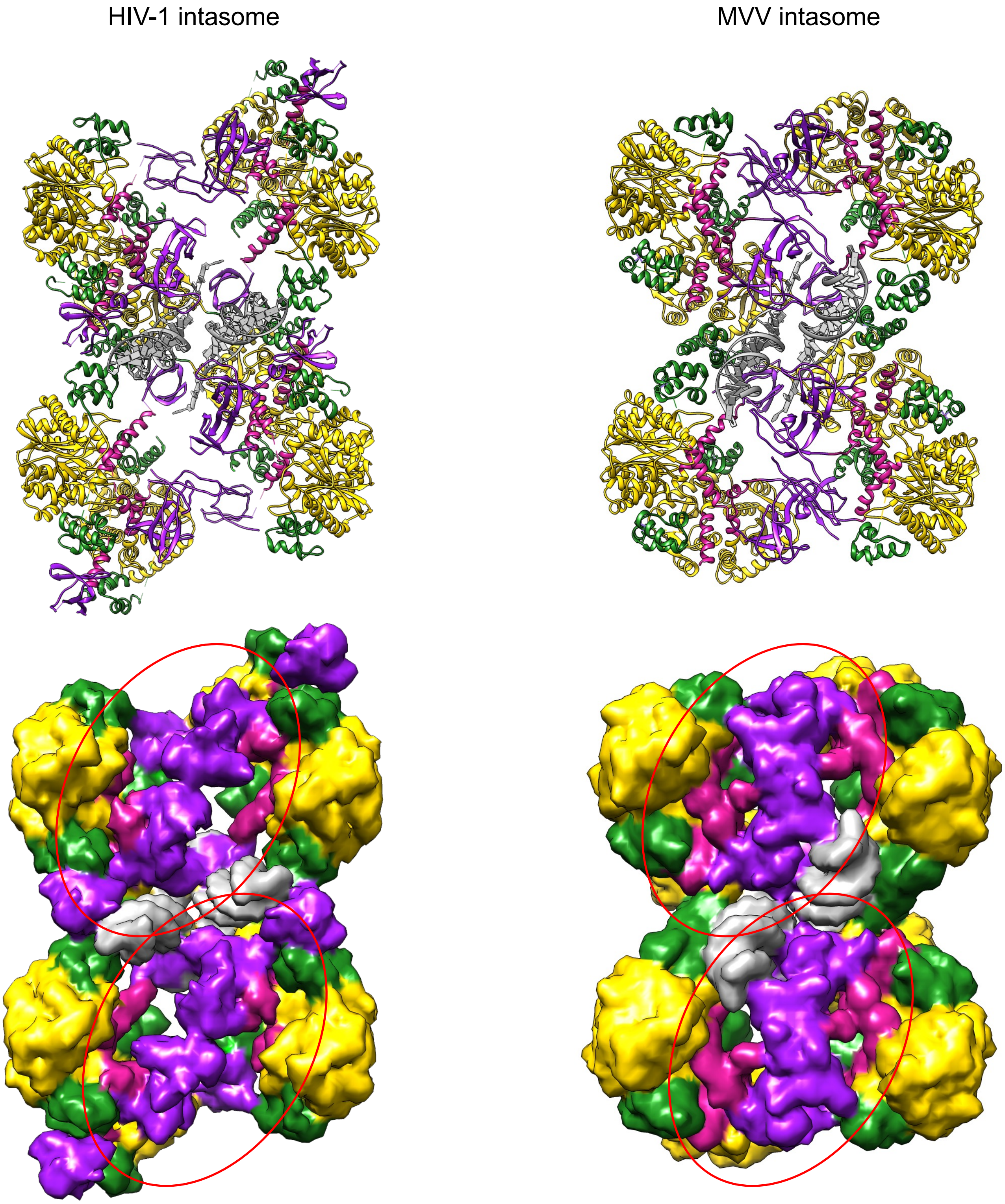

Supplementary Figure 13

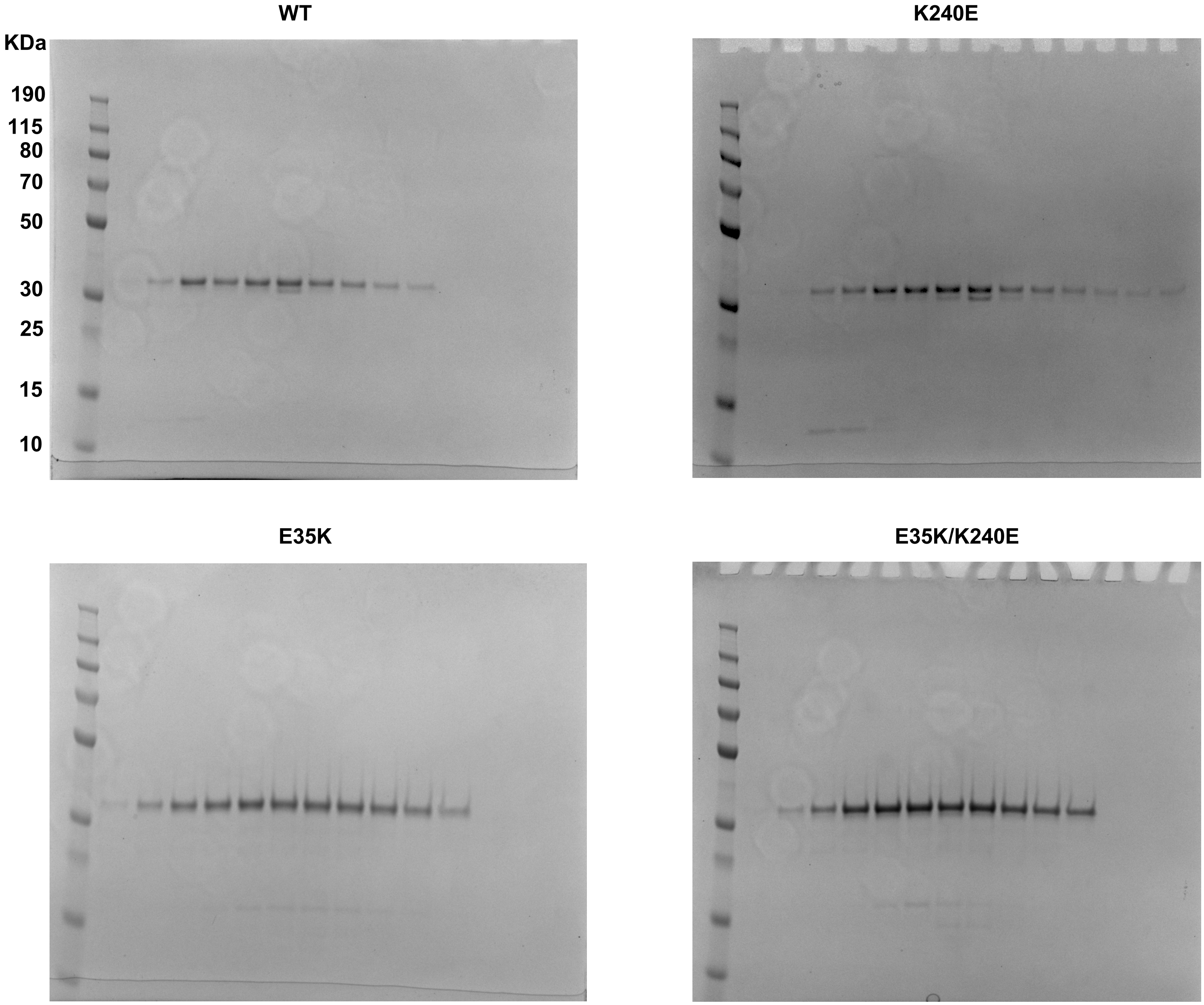

Supplement: Supplementary file 1 [file NIHPP2024.01.26.577436V1-supplement-1.pdf]
